# Penetrative and non-penetrative interaction between Laboulbeniales fungi and their arthropod hosts

**DOI:** 10.1038/s41598-021-01729-x

**Published:** 2021-11-12

**Authors:** Ana Sofia P. S. Reboleira, Leif Moritz, Sergi Santamaria, Henrik Enghoff

**Affiliations:** 1 Departamento de Biologia Animal, Faculdade de Ciências, Centre for Ecology, Evolution and Environmental Changes (cE3c), Universidade de Lisboa, Lisbon, Portugal; 2 Natural History Museum of Denmark, University of Copenhagen, 2100 København Ø, Denmark; 3 Zoological Research Museum Alexander Koenig, Leibniz Institute for Animal Biodiversity, Adenauerallee 160, 53113 Bonn, Germany; 4 Institute of Evolutionary Biology and Ecology, University of Bonn, An der Immenburg 1, 53121 Bonn, Germany; 5 Unitat de Botànica, Departament de Biologia Animal, de Biologia Vegetal I d’Ecologia, Facultat de Biociències, Universitat Autònoma de Barcelona, 08193 Cerdanyola del Vallès, Barcelona Spain

**Keywords:** Entomology, Ecophysiology, Fungal biology, Fungal ecology, Fungal host response, Fungal pathogenesis

## Abstract

Laboulbeniales are a highly specialized group of fungi living only on arthropods. They have high host specificity and spend their entire life-cycle on an arthropod host. Taxonomic characters of Laboulbeniales are based on the architecture of the cells of the parenchymal thallus, i.e. the visible part of the fungus outside the host. The extent of the fungus spreading inside the host—the haustorium—remains largely unknown. The attachment to the arthropod host is fundamental to understand the fungus-animal interaction, but how this truly occurs is unclear. Recent evidences question the strictly parasitic life-style of Laboulbeniales. We used micro-computed tomography (µCT) and 3D reconstructions to visualize, for the first time, the complete structure of Laboulbeniales species in situ on their hosts. We compared the haustoriate species, *Arthrorhynchus nycteribiae* on an insect host to the non-haustoriate species, *Rickia gigas* on a millipede host. Our results confirm that some Laboulbeniales species are ectoparasitic and have a haustorial structure that penetrates the host’s cuticle, while others are ectobionts and are only firmly attached to the host’s cuticle without penetrating it. The presence and the morphology of the haustorium are important traits for Laboulbeniales evolution, and key factors for future understanding of host dependence and specificity.

## Introduction

Parasitism is defined as the relationship between two organisms where one organism, the parasite, benefits while the other organism, the host, is harmed by the relationship^1^. Typically, the parasite extracts nourishment from the host and thereby reduces its fitness^1^. A parasite itself can be infected with another parasite, which defines the latter as a hyperparasite^2^. Understanding parasitism, especially hyperparasitism, requires a complex multidisciplinary approach involving ecology, evolution and behaviour of the three participants in the interaction^2–5^.

Laboulbeniales have long been considered ectoparasites of living arthropods, where they can be found on the external surface of their cuticle^6,7^. Laboulbeniales hosts must combine important properties: (i) successive generations of adult hosts should overlap in time because transmission occurs mainly during copulation, because the vast majority does not live on eggs or larval stages of their host; (ii) their populations must be large and stable; and (iii) they must inhabit moist environments^8^. Studies on Laboulbeniales have mostly been taxonomic, with a very recent emergence of phylogenetic analysis^9–13^, and a few recent studies have provided insights into the interaction of Laboulbeniales and their hosts, and the environment^2,14–17^.

Morphological studies on Laboulbeniales have focused on the external part of the fungus, the thallus. A recent paper on the histopathology of the genus *Rickia* started a debate on the absence of the haustorial structures in some genera of Laboulbeniales^18^, whether this is a secondary loss remains unknown.

We investigate the presence and reveal the structure of Laboulbeniales haustoria in situ in their hosts, using the novel visualization technique based on micro-CT, and also on scanning electron microscopy (SEM).

## Results

### Micro-computed tomography (µCT)

**Figure 1:**
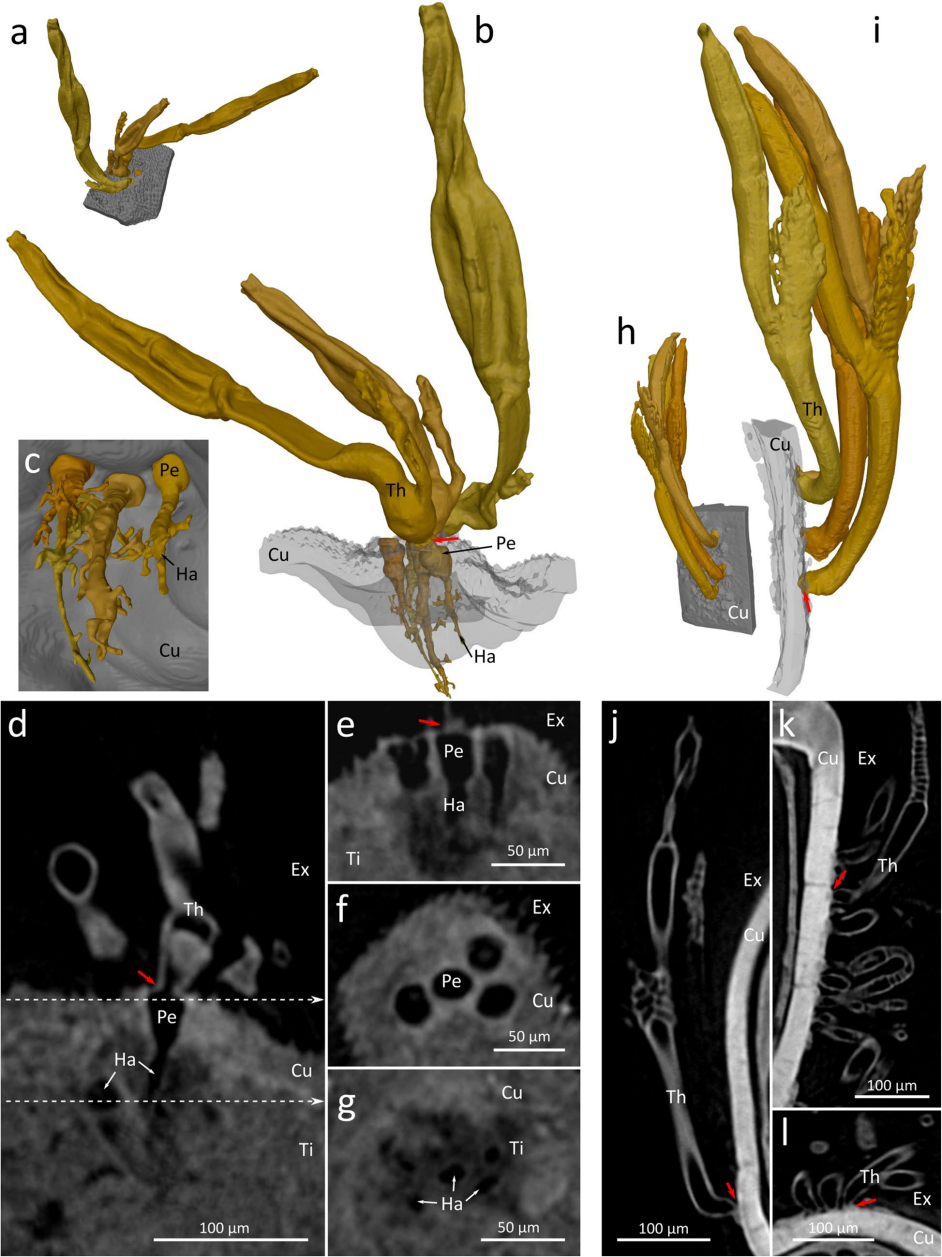
Comparison of the haustoriate Laboulbeniales *Arthrorhynchus nycteribiae* (**a–g**) on the abdomen of the batfly *Penicillidia conspicua* (SR-Nyct85) and the non-haustoriate *Rickia gigas* on the leg of the millipede *Tropostreptus hamatus* (**h–l**). (**a–c,i,h**) Segmentation based on µCT data. (**d–g,j–l**) Virtual sections based on µCT data. (**a**) External view. (**b**) Penetration of the hosts cuticle (transparent). (**c**) Haustorium, internal view. (**d**) Transverse section trough the fungi and the host’s cuticle. (**e**) Transverse section trough the host’s cuticle underneath the base of the thallus. (**f**) Section trough the host’s cuticle underneath the base of the thallus, plane as indicated in (**d**). (**g**) Section trough the host’s tissue and haustorium, plane as indicated in (**d**). (**h**) External view. (**i)** Penetration of the hosts cuticle (transparent). (**j**) Transverse section trough the fungi and the host’s cuticle. (**k**) longitudinal section through the host’s leg. (**l**) Cross section trough the host’s leg. *Cu* host’s cuticle, *Ex* exterior, *Ha* haustorium, *Pe* penetration of cuticle, *Th* thallus, *Ti* host’s tissue. Red arrows indicate the base (foot) of the thalli. Figure plate prepared with Gimp 2.10.6 (GIMP Development Team, https://www.gimp.org) and Inkscape 1.0 (Inkscape Developers, https://inkscape.org).

A 3D reconstruction based on µCT of *Arthrorhynchus nycteribiae* on a male *Penicillidia conspicua*, shows host cuticle with attached thalli, attached to an intersegment membrane (Fig. 1 and Supplementary Video S1), and a group of four thalli (of which one is broken at the base) of *Arthrorhynchus nycteribiae.* At the base of each thallus the cuticle (with a diameter of 25–30 µm) is penetrated by a circular hole with a diameter of 22–28 µm (n = 4). From this penetration a cylindrical lumen, which we interpret as the haustorium extends 45–71 µm (n = 4) into the host’s tissue. Inside the host’s body cavity this structure, the haustorium, tapers distally and gives rise to several side branches. In contrast, at the base of the four thalli of *Rickia gigas* shown in (Fig. 1 and Supplementary Video S2), no such penetration into the host’s cuticle is visible in the µCT-data.

### Scanning electron microscope (SEM)

**Figure 2:**
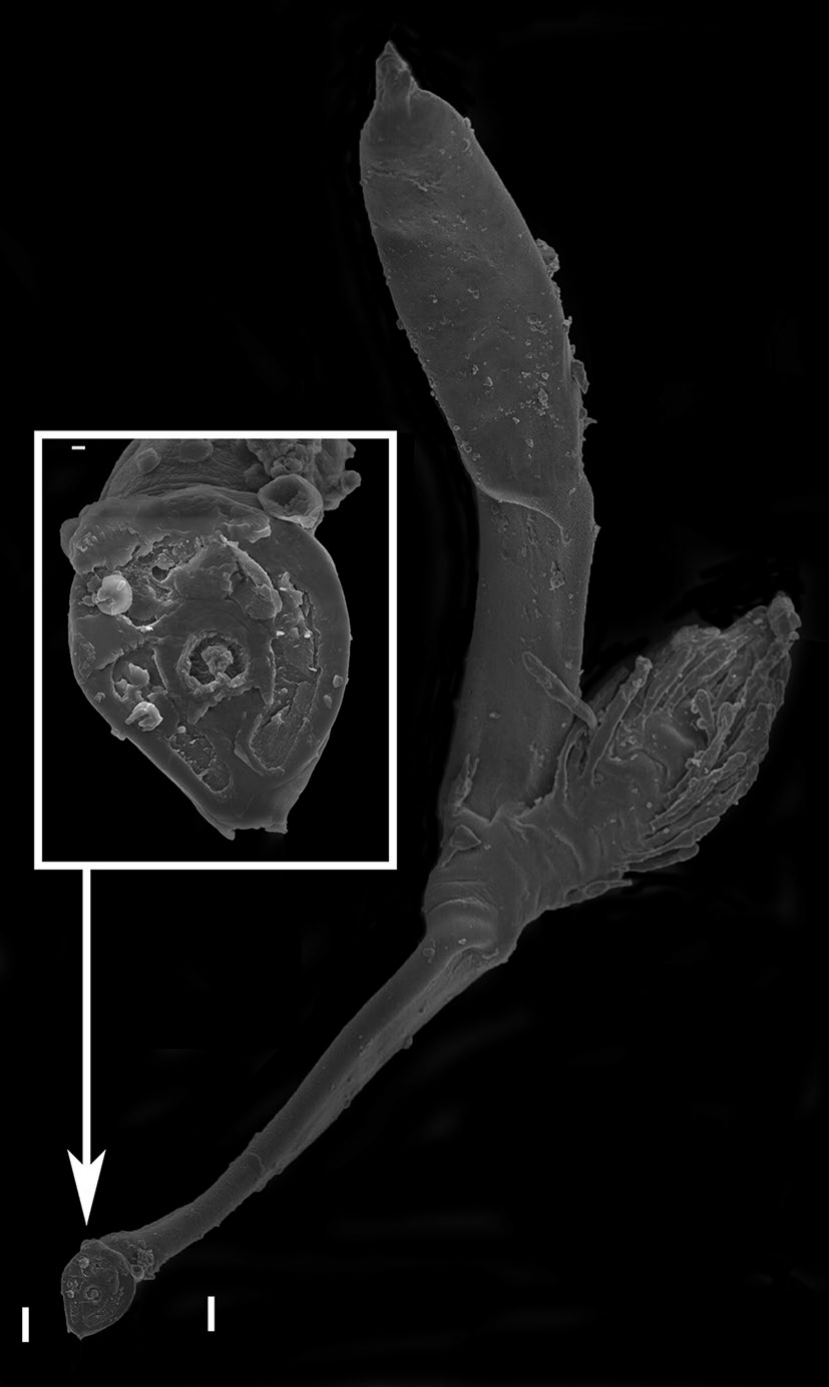
*Rickia gigas* on the cuticle of the millipede *Tropostreptus hamatus,* SEM image, thallus and detail of the inner part of the foot. Scale bars: 10 μm in overview, 1 μm in detail image.

**Figure 3:**
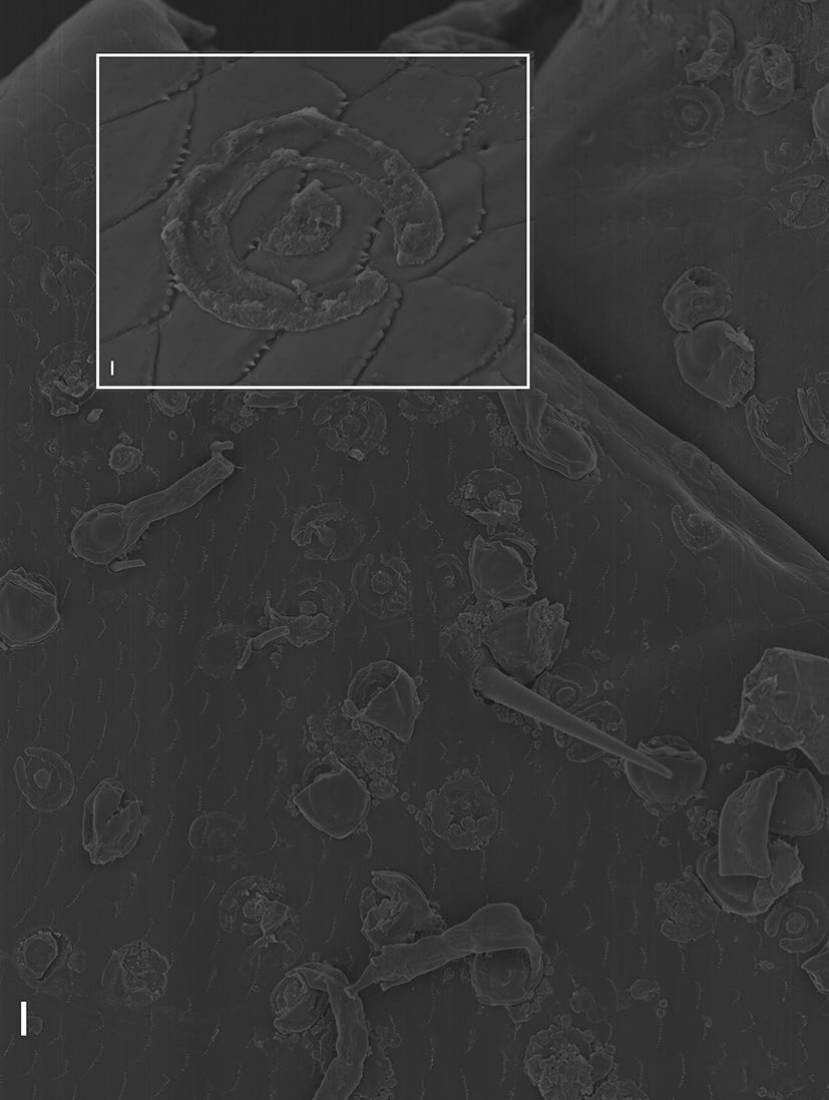
SEM image of detached *Rickia gigas* on the cuticle of the millipede *Tropostreptus hamatus*. Scale bars: 10 μm in overview image, 1 μm in detail image.

We here present SEMs of the “gigantic” non-haustoriate species *Rickia gigas*. When a thallus of *R. gigas* is separated from the host cuticle, an horseshoe-shaped wall remains on the host, surrounding an amorphous structure in the middle (Fig. 2). This structure is mirrored by the foot. On the underside of the foot of the fungus, a similar horseshoe-shaped rim, or impression of a wall, is evident. Within this outer rim/wall a smaller circular structure is evident, which surrounds an amorphous substance in the middle. The inner circle attaches to the foot’s outer margin in a position corresponding to the opening in the horseshoe-shaped ring wall (Fig. 3). SEM images of *Arthrorhynchus—*a Laboulbeniales species with haustoria—were previously shown^2^.

## Discussion

The micro-CT results from *Arthrorhynchus* agree perfectly with the previously known light microscope and transmission electron microscope images^2^. This emphasizes that microtomography is a good technique to visualize the type of fungal attachment to the host and especially the penetration of the cuticle, apart from the study of thallus in amber fossils^17^. As Jensen et al. (2019) demonstrated the presence of a haustorium in *Arthrorhynchus* using scanning electron microscopy, we are confident that the lack of penetration and haustorium in *Rickia* found by micro-CT is real. This is also in agreement with results from the scanning electron microscopical investigation of the attachment sites of *R. gigas*, which exhibits no indication of penetration and are very similar to those of *R. wasmannii* previously shown^18^.

Despite the absence of a haustorium, and hence without any obvious means of obtaining nutrition, *Rickia gigas* is quite a successful fungus, being often abundant on several species of Afrotropical millipedes of the family Spirostreptidae^10^. It was originally described from *Archispirostreptus gigas,* and *Tropostreptus* (= ‘Spirostreptus’) *hamatus*^20^*,* and was subsequently reported from several other *Tropostreptus* species^19^.

A further challenge for Laboulbeniales growing on millipedes is that infected millipedes, in some species even adults, may moult, shedding the exuviae with the fungus, as has been observed by us on an undescribed *Rickia* species on a millipede of the genus *Spirobolus* (family Spirobolidae).

The question of how non-haustoriate Laboulbeniales obtain nutrients has been discussed by several authors^18^, including staining experiments using fungi of the non-haustoriate genus *Laboulbenia* on various beetles^21^. Whereas the surface of the main thallus was almost impenetrable to the dye applied (Nile Blue), the smaller appendages could sometimes be penetrated^21^. The dye injection into the beetle elytra upon which the fungi were sitting, actually spread from the elytron into the fungus, thus indicating that in spite of the lack of a haustorium, the fungus is able to extract nutrients from the interior of its host^21^.

Such experiments have not been performed on *Rickia* species, but the possibility that nutrients may pass from the host into the basis of the fungus cannot be excluded. For this genus, or at least *R. gigas*, there may, however, be an alternative way to obtain nutrients: the small opening in the circular wall by which the thallus is attached to the host may allow nutrients from the surface of the millipede or from the environment to seep into the foot of the fungus. However, further experiments are needed in order to evaluate this hypothesis. Moreover, we should not exclude a potential role of primary and secondary appendages in Laboulbeniales nutrition, as we still do not understand exactly their functional role on the fungus life cycle^11^.

The predominant position of the Laboulbeniales on the host might be related to the absence or presence of a haustorium. Thus, the haustoriate species of the genus *Arthrorhynchus* are most frequently encountered in large numbers on the arthrodial membranes of the host’s abdomen, although some thalli are found on legs^2,22^. At the arthrodial membranes the cuticle is more flexible and therefore might be easier to penetrate by a parasite. Furthermore, most tissues providing/storing nutrition (e.g., fat body) are located within the abdomen. In contrast, non-haustoriate fungi as are often located on more stiff and sclerotized body-parts like the genus *Rickia* on the legs or body-rings of millipedes^7,20,23^ or the genus *Laboulbenia* on the elytra of beetles^21,24^. A reason for this might be that the non-haustoriate forms, which are only superficially attached to the host need a more or less smooth surface for adherence and can easily become detached from a flexible surface, which is movable in itself, like the arthrodial membrane, while the haustoriate forms are firmly anchored within the hosts’ cuticle.

Whereas the vast majority of the more than 2000 described species of Laboulbeniales show no sign of host penetration, haustoria have been reported from some other genera^18^, including *Trenomyces* parasitizing bird lice^25,26^, *Hesperomyces* growing on coccinellid beetles and *Herpomyces* on cockroaches (formerly a Laboulbeniales and now in the order Herpomycetales^10^), with pernicious consequences on the hosts’ fitness^18,27^. Micro-CT studies on these genera could help to understand the host penetration. In order to fully understand how Laboulbeniales obtain nourishment, although other approaches are, also needed—for the time being it remains a mystery how the non-haustoriate Laboulbeniales sustain themselves.

## Methods

### Specimens used

All specimens were obtained from the collection of the Natural History Museum of Denmark. *Arthrorhynchus nycteribiae* (Peyr.) Thaxt. on *Penicillidia conspicua* Speiser, 1901, a male bat fly, infected on the dorsal part of the abdomen, 21.07.2018, Igrejinha de Soídos, Algarve, Portugal, L. Rodrigues & S. Reboleira leg. (ref. SR-Nyct85); *Rickia gigas* Santam., Enghoff & Reboleira on *Tropostreptus hamatus* (Demange, 1977), a male millipede heavily infected on the legs, Udzungwa Mountains Natural Park, Sanje Chini camp, 598 m, 17-20.01.2014, Thomas Pape leg.

Specimens were initially examined under a binocular stereomicroscope Leica M165C, and measurements were made with the software Leica Application Suite V4.12. Fungal thalli were dissected and mounted on temporary slides in glycerine for morphological taxonomic study, following the standard methodology^8^, under light microscopy in a Leica DM2500 microscope with Differential Interference Contrast (DIC).

### Micro-computed tomography (µCT)

Specimens for micro-computed tomography (µCT) were transferred to 100% ethanol, stained for 24 h in 3% Iodine-solution and washed subsequently with 100% ethanol. These specimens were then critical point dried (CPD) with a Leica EM CPD 300, mounted in a pipette tip and scanned using a SKYSCAN 1272 (Bruker microCT) with the following scanning parameters: source voltage = 25 kV, source current = 130 μA, exposure = 4000 ms, rotation of 360° in rotational steps of 0.2°, frame averaging = 7, random movement = 15, filter = none, pixel size = 0.8 µm. Post-alignment, ring-artefact reduction, beam-hardening correction and reconstruction was performed in NRecon 1.7.1.6 (Bruker microCT). The image stack was modified using Fiji ImageJ 1.50e^28^ (https://www.imagej.net). Volume rendering and measurements were performed in Drishti Version 2.6.3^29^ (https://www.github.com/nci/drishti), and segmentation of the thallus and the haustorium was done in ITKSnap 3.8.0^30^ (http://www.itksnap.org), within the host the lumen supposedly created by the haustorium was segmented. Smoothing, rendering and animation was performed in MeshLab 1.3.3^31^ (https://www.meshlab.net) and Blender 2.77 (Blender Foundation; https://www.blender.org). Figures were prepared in GIMP 2.10.6 (GIMP Development Team; https://www.gimp.org) and Inkscape 1.0 (Inkscape Developers; https://inkscape.org). The generated µCT-data is deposited on Zenodo under doi: 10.5281/zenodo.4737626.

### Scanning electron microscopy (SEM)

Specimens for SEM were transferred to 100% ethanol, critical point-dried in a Tousimis Autosamdri 815 Series A critical point dryer, mounted on an aluminium stub, coated with platinum/palladium and studied under a JEOL JSM-6335F scanning electron microscope.

## Supplementary Information

Supplementary Legends.

Supplementary Video 1.

Supplementary Video 2.

## References

[1] Sulivan, D. Hyperparasitism. in *Encyclopedia of Insects*. 486–488. (Academic Press, 2009)

[2] Jensen, K. M. *et al.* Hyperparasitism in caves: Bats, bat flies and ectoparasitic fungus interaction. *J. Invertebr. Pathol.* **166**, 107206. 10.1016/j.jip.2019.107206 (2019) 10.1016/j.jip.2019.107206 31152770

[3] Haelewaters D (2017). Parasites of parasites of bats: Laboulbeniales (Fungi: Ascomycota) on bat flies (Diptera: Nycteribiidae) in central Europe. Parasit. Vectors..

[4] Haelewaters D, Page R, Pfliegler WP (2018). Laboulbeniales hyperparasites (Fungi, Ascomycota) of bat flies: Independent origins and host associations. Ecol. Evol..

[5] Haelewaters D (2019). Studies of Laboulbeniales on *Myrmica* ants (IV): Host-related diversity and thallus distribution patterns of *Rickia wasmannii*. Parasite.

[6] Santamaria S, Enghoff H, Gruber J, Reboleira ASPS (2017). First Laboulbeniales from harvestmen: The new genus *Opilionomyces*. Phytotaxa.

[7] Santamaria S, Enghoff H, Reboleira ASPS (2018). New species of *Troglomyces* and *Diplopodomyces* (Laboulbeniales, Ascomycota) from millipedes (Diplopoda). Eur. J. Taxon..

[8] Reboleira ASPS, Enghoff H, Santamaria S (2018). Novelty upon novelty visualized by rotational scanning electron micrographs (rSEM): Laboulbeniales on the millipede order Chordeumatida. PLoS ONE.

[9] Goldmann L, Weir A (2018). Molecular phylogeny of the Laboulbeniomycetes (Ascomycota). Fungal Biol..

[10] Haelewaters D, Pfliegler W, Gorczak M, Pfister D (2019). Birth of an order: Comprehensive molecular phylogenetic study excludes *Herpomyces* (Fungi, Laboulbeniomycetes) from Laboulbeniales. Mol. Phylogenet. Evol..

[11] Haelewaters D, Blackwell M, Pfister DH (2021). Laboulbeniomycetes: Intimate fungal associates of arthropods. Annu. Rev. Entomol..

[12] Blackwell M, Haelewaters D, Pfister DH (2020). Laboulbeniomycetes: Evolution, natural history, and Thaxter’s final word. Mycologia.

[13] Sundberg H, Kruys Å, Bergsten J, Ekman S (2018). Position specificity in the genus *Coreomyces* (Laboulbeniomycetes, Ascomycota). Fungal Syst. Evol..

[14] Szentiványi T (2018). Laboulbeniales (Fungi: Ascomycota) infection of bat flies (Diptera: Nycteribiidae) from *Miniopterus schreibersii* across Europe. Parasit. Vectors.

[15] Szentiványi T (2019). Climatic effects on the distribution of ant- and bat fly-associated fungal ectoparasites (Ascomycota, Laboulbeniales). Fungal Ecol..

[16] Csősz S (2021). Ectoparasitic fungi *Rickia wasmannii* infection is associated with smaller body size in *Myrmica* ants. Sci. Rep..

[17] Perreau M, Haelewaters D, Tafforeau P (2021). A parasitic coevolution since the Miocene revealed by phase-contrast synchrotron X-ray microtomography and the study of natural history collections. Sci. Rep..

[18] Tragust S, Tartally A, Espadaler X, Billen J (2016). Histopathology of Laboulbeniales (Ascomycota: Laboulbeniales): Ectoparasitic fungi on ants (Hymenoptera: Formicidae). Myrmecol. News.

[19] Enghoff H (2017). A new East African genus of spirostreptid millipedes (Diplopoda, Spirostreptida, Spirostreptidae), with notes on their fungal ectoparasite *Rickia gigas*. Zootaxa.

[20] Santamaria S, Enghoff H, Reboleira ASPS (2016). Hidden biodiversity revealed by collections-based research—Laboulbeniales in millipedes: Genus *Rickia*. Phytotaxa.

[21] Scheloske HW (1969). Beiträge zur Biologie, Ökologie und Systematik der Laboulbeniales (Ascomycetes) unter besonderer Berücksichtigung des Parasit-Wirt-Verhältnisses. Parasitol. Schriftenreihe.

[22] Blackwell M (1980). Incidence, host specificity, distribution and morphological variation in *Arthrorhynchus nyctyeribiae* and *Arthrorhynchus eucampsipodae* (Laboulbeniomycetes). Mycologia.

[23] Santamaria S, Enghoff H, Reboleira ASPS (2014). Laboulbeniales on millipedes: The genera *Diplopodomyces* and *Troglomyces*. Mycologia.

[24] Santamaria S (2006). New or interesting Laboulbeniales (Fungi, Ascomycota) from Spain, V. Nova Hedwigia.

[25] Meola S, Devaney J (1976). Parasitism of mallophaga by *Trenomyces histophtorus*. J. Invertebr. Pathol..

[26] Meola S, Tavares II (1982). Ultrastructure of the haustorium of *Trenomyces histophthorus* and adjacent host cells. J. Invertebr. Pathol..

[27] Szentiványi T (2020). Effects of fungal infection on the survival of parasitic bat flies. Parasites Vectors.

[28] Schindelin J (2012). Fiji: an open-source platform for biological-image analysis. Nat. Methods.

[29] Limaye, A. Drishti: A volume exploration and presentation tool. in *Developments in X-Ray Tomography VIII*. (Stock, S.R. Ed.). *Proceedings of SPIE*. Vol. 8506. 85060. 10.1117/12.935640 (2012).

[30] Yushkevich PA (2006). User-guided 3D active contour segmentation of anatomical structures: Significantly improved efficiency and reliability. Neuroimage.

[31] Cignoni, P. *et al.* Meshlab: An open-source mesh processing tool. in *Eurographics Italian chapter conference*. 129–136 (2008).

