# Supplementary material for: Penetrative and non-penetrative interaction between Laboulbeniales fungi and their arthropod hosts
Source: Sci Rep. 2021 Nov 12;11:22170. doi: 10.1038/s41598-021-01729-x (PMC8589835; doi:10.1038/s41598-021-01729-x)
Supplement: Supplementary file 1 — Supplementary Legends. [file 41598_2021_1729_MOESM1_ESM.docx]

**Penetrative and non-penetrative interaction between Laboulbeniales fungi and their arthropod hosts**

**Ana Sofia P.S. Reboleira^1,2*^; Leif Moritz^3,4^; Sergi Santamaria^5^; Henrik Enghoff^2^**

^1^ Centre for Ecology, Evolution and Environmental Changes (cE3c), and Departamento de Biologia Animal, Faculdade de Ciências, Universidade de Lisboa, Lisbon, Portugal

^2^ Natural History Museum of Denmark, University of Copenhagen, 2100 København Ø, Denmark

^3^ Zoological Research Museum Alexander Koenig, Leibniz Institute for Animal Biodiversity, Adenauerallee 160, D-53113, Bonn, Germany

^4^ Institute of Evolutionary Biology and Ecology, University of Bonn, An der Immenburg 1, D-53121 Bonn, Germany

^5^ Unitat de Botànica, Departament de Biologia Animal, de Biologia Vegetal i d'Ecologia, Facultat de Biociències, Universitat Autònoma de Barcelona, 08193-Cerdanyola del Vallès (Barcelona), Spain

^*^Corresponding author: asreboleira@fc.ul.pt

**Supplementary file section**

**Supplementary video 1:** Laboulbeniales *Arthrorhynchus nycteribiae* (Peyr.) Thaxt. penetrating the cuticle of a male bat fly *Penicillidia conspicua*. Video (avi) of 3D segmentation based on µCT data, rendered in Blender 2.77 (Blender Foundation; <https://www.blender.org>).

**Supplementary video 2:** Laboulbeniales *Rickia gigas* Santam., Enghoff & Reboleira, 2016 on the millipede *Tropostreptus hamatus* (Demange, 1977). Video (avi) of 3D segmentation based on µCT data, rendered in Blender 2.77 (Blender Foundation; <https://www.blender.org>).
